# Synthesis and Crystal Structure of the Bioinorganic Complex [Sb(Hedta)]*·*2H_2_O

**DOI:** 10.1155/2014/461605

**Published:** 2014-02-13

**Authors:** Di Li, Guo-Qing Zhong

**Affiliations:** State Key Laboratory Cultivation Base for Nonmetal Composite and Functional Materials, School of Material Science and Engineering, Southwest University of Science and Technology, Mianyang 621010, China

## Abstract

The antimony(III) complex [Sb(Hedta)]*·*2H_2_O was synthesized with ethylenediaminetetraacetic acid (H_4_edta) and antimonous oxide as main raw materials in aqueous solution. The composition and structure of the complex were characterized by elemental analysis, infrared spectra, single crystal X-ray diffraction, X-ray powder diffraction, thermogravimetry, and differential scanning calorimetry. The crystal structure of the antimony(III) complex belongs to orthorhombic system, space group Pna2(1), with cell parameters of *a* = 18.4823(18) Å, *b* = 10.9408(12) Å, *c* = 7.3671(5) Å, *V* = 1489.7(2) Å^3^, *Z* = 4, and *D*_*c*_ = 1.993 g cm^−3^. The Sb(III) ion is five-coordinated by two amido N atoms and three carboxyl O atoms from a single Hedta^3−^ ligand, forming a distorted trigonal bipyramid geometry. The thermal decomposition processes of the complex include dehydration, oxidation, and pyrolysis of the ligand, and the last residue is Sb_2_O_3_ at the temperature of 570°C.

## 1. Introduction

Many of the antimony(III) compounds have been mainly used in the clinic medicine because of their medicinal utility and biological activity. The aminopolycarboxylate complexes of antimony(III) with effective antimicrobial and anticancer activity are widely applied to the treatment of a variety of microbial infections including leishmaniasis, diarrhea, peptic ulcers, and helicobacter pylori, and so forth [1, 2]. The ethylenediaminetetraacetic acid as one of the multicarboxylate ligands possesses diverse functional groups such as N-donor and O-donor. When a metal ion is in the center position, the ligand is always allowed to bend and to rotate, and one can easily imagine the structural diversity of new synthesizable materials [3]. In fact, the design and synthesis of metal-organic frameworks (MOFs) is not only because of their fascinating structures and topological novelty, but also because of their existing potential applications as functional materials in gas storage, heterogeneous catalysis, chemical separations, and microelectronics [4–11]. So, the ethylenediaminetetraacetic acid (H_4_edta) as ligand is a good choice for building a diversified structure; it is not only a plurality of coordination sites but also an inexpensive and relative safe substance, which can remove toxic heavy metals, reduce oxidative stress, and increase waste excretion [12, 13]. In clinical practice, the chelation therapy with H_4_edta can prevent cancer and catalytic reactive oxygen species, such as cardiovascular and arteriosclerotic heart disease [14–16]. So, it is widely used in the pharmaceutical and biological aspects.

As we know, the main group elements do not easily form complexes with organic ligands because of the special properties. But, owing to the presence of a lone pair of electrons, showing stereochemical behavior, the antimony complexes with aminopolycarboxylate ligands have attracted people's interests [17]. Because the inorganic salts of antimony(III) have considerable toxicity, so the new biological activity and medicinal function complexes of the main group element antimony are received more attention [18, 19]. Besides, some antimony(III) compounds have been used as antiparasitic agents, and exhibit significant functions as biocides, fungicides, antioxidants, and potential therapeutic agents. For example, some antimony compounds are used for treatment of leishmaniasis, various species of the protozoan leishmania virus and cancer [20–25].

In recent years, owing to the development of aminopolycarboxylic acid complexes of antimony(III) in medicine, the complex of antimony with multicarboxylate ligands has received more and more attention. Herein, we report the syntheses of the title complex [Sb(Hedta)]·2H_2_O. The composition and crystal structure of the complex have been characterized by elemental analysis, single crystal X-ray diffraction, XRD, FTIR, and TG-DSC.

## 2. Experimental

### 2.1. Materials and General Methods

All the chemicals used in the experiments were analytical reagent as received from commercial sources and without further purification. The ethylenediaminetetraacetic acid and antimonous oxide were purchased from Shanghai Reagent Industry.

The antimony was determined by a Thermo X-II inductively coupled plasma mass spectrometer. The content of carbon, hydrogen, and nitrogen in the complex was measured by a Vario EL CUBE elemental analyzer. The IR spectra were obtained with a Perkin-Elmer Spectrum One-spectrometer in the range of 225–4000 cm^−1^ using KBr pellets. The thermogravimetric analysis of the metal complex was performed by an SDT Q600 thermogravimetric analyzer, and the measurement was recorded from 30 to 800°C at the heating rate of 10°C min^−1^ under air flow of 100 mL min^−1^. The X-ray powder diffraction was performed using a D/max-II X-ray diffractometer, Cu K_*α*_ radiation (*λ* = 0.154056 nm, step width: 2*θ* = 0.2°, scan speed: 8°/min).

### 2.2. Synthesis of the Complex [Sb(Hedta)]·2H_2_O

The title complex was obtained by aqueous solution synthesis. First, 1.46 g (5 mmol) ethylenediaminetetraacetic acid was solubilized in 200 mL boiling distilled water. Then, 0.875 g (3 mmol) antimonous oxide was gradually added to the above solution, stirring and maintaining the temperature at 90°C. After four hours, when the pH was about 2-3, the reaction was stopped. The unreacted antimonous oxide was filtered. The colorless filtrate was slowly concentrated to 100 mL. The concentrated solution was placed at room temperature for about one week, and the colorless flaky crystals of the antimony(III) complex was obtained. The yield was about 82%. Anal. Calcd for the title complex, SbC_10_H_17_N_2_O_10_(%): Sb, 27.24; C, 26.87; N, 6.27; H, 3.83. Found (%): Sb, 27.11; C, 26.68; N, 6.19; H, 3.74.

### 2.3. X-Ray Diffraction Crystallography

The appropriate crystals were cut from larger crystals and mounted on a Bruker Smart Apex II CCD diffractometer with graphite monochromated Mo K_*α*_ radiation (*λ* = 0.71073 Å). The data were collected at 298(2) K. A colorless and transparent crystal with dimensions 0.46 mm × 0.40 mm × 0.35 mm was mounted on a glass fiber. Diffraction data were collected in *ω* mode in the range of 2.89°–25.02°. The structure was solved by direct methods SHELXS-97 and refined by full-matrix least-squares using SHELXL-97 [26, 27]. All nonhydrogen atoms were obtained from the difference Fourier map and full-matrix least-squares refinements on *F* ^2^ were carried out with anisotropic thermal parameters. Hydrogen atoms of the ligand were generated geometrically. The structure refinement parameters for the title complex are given in Table 1, and the crystallographic data are deposited with the Cambridge Crystallographic Data Centre under deposition number CCDC 953660.

## 3. Results and Discussion

### 3.1. X-Ray Crystal Structure Analysis

**Figure 1:**
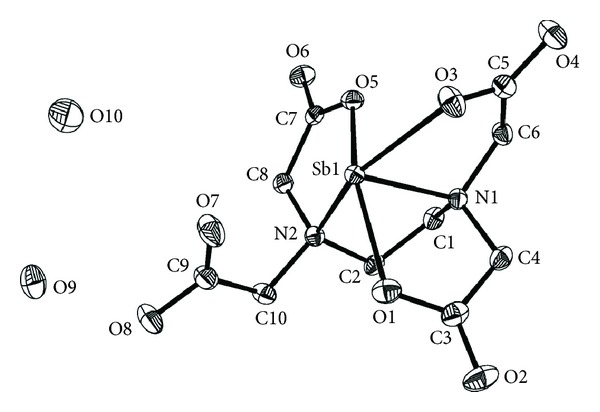
The molecular structure of the title complex.

**Figure 2:**
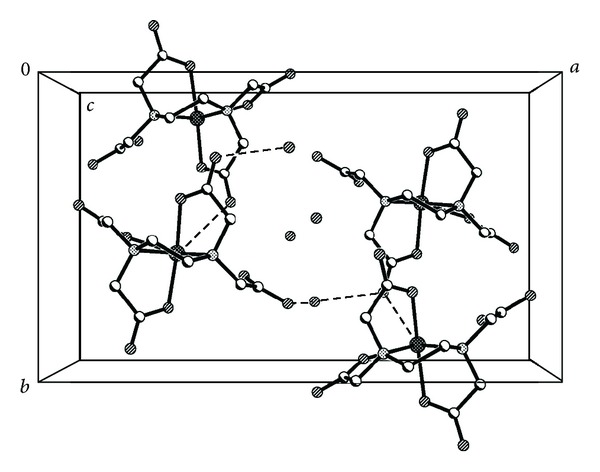
Crystal packing diagram of the title complex.

The single crystal X-ray diffraction analysis reveals that the complex [Sb(Hedta)]·2H_2_O crystallises in the orthorhombic system with space group Pna2(1). Crystallographic data and structure refinement parameters for the title complex are given in Table 1, and the selected bond distances and angles are shown in Table 2. The key fragments of the structures and the atom numbering are shown in Figure 1, and the crystal packing diagram of the complex is shown in Figure 2. The asymmetric unit of the complex consists of one Hedta^3−^, one trivalent antimony ion, and two crystalline water molecules. Sb(III) ion for plane forms a distorted trigonal bipyramidal configuration. The Sb(III) ion is five-coordinated by three carboxyl oxygen atoms (O1, O3, and O5) and two nitrogen atoms (N1 and N2) from the same Hedta^3−^ ligand. Therefore, a carboxyl group (O7 and O8) and all water molecules in the title complex are not coordinated. The bond lengths of Sb1–O1, Sb1–O3, and Sb1–O5 are in the range of 2.182–2.243 Å, and it belongs to the typical distance of the aminopolycarboxylate complexes of antimony(III) [28]. But, with respect to the bond lengths of C–O, there are two types of carboxyl groups in the complex. The C5(7)–O3(5) is longer than C5(7)–O4(6), about 0.04 Å, like the usual ionized carboxyl group. But, the C3(9)–O1(8) is longer than C3(9)–O2(7), about 0.08 Å, which is comparable with the value of a free carboxylic acid, and this type of coordination seems to cause the especially strong acidity of Sb-(Hedta). The bond angles of N1–Sb1–O3, N1–Sb1–O5, N1–Sb1–N2, and N1–Sb1–O1 are almost similar; if the Sb1–N1 is perpendicular to the equator plane, they look like an inverted umbrella, and the Sb–N bond lengths are 2.336(5) Å and 2.402(5) Å, and the Sb–O band lengths are Sb1–O1 = 2.182(4) Å, Sb1–O3 = 2.243(4) Å, and Sb1–O5 = 2.199(4) Å, respectively.

**Figure 3:**
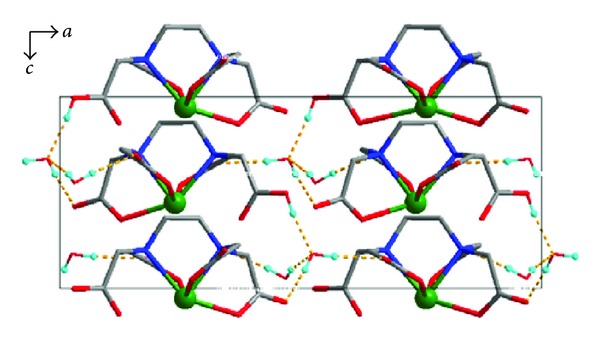
Packing diagram of the title complex showing H bonding along the *b* axis.

In Tables 2 and 3, there are three types of hydrogen bonds in the crystal of the Sb(III) complex, and they include the weak hydrogen bonds between the crystalline water molecules (O10–H10E ⋯ O9, 2.950 Å), the crystalline water and the oxygen atoms of the carbonyl groups (O9–H9C ⋯ O4, 2.702 Å; O9–H9D ⋯ O6, 2.783 Å, and O10–H10F ⋯ O2, 2.930 Å), and the strong hydrogen bonds between the crystalline water and the oxygen atoms of the hydroxyl groups (O8–H8 ⋯ O9, 2.556 Å). In Figure 3, because there are a lot of crystal water molecules in the complex, the interstitial water molecules and the carboxyl oxygen atoms will form hydrogen bonds, which make the structure more stable.

### 3.2. X-Ray Powder Diffraction

**Figure 4:**
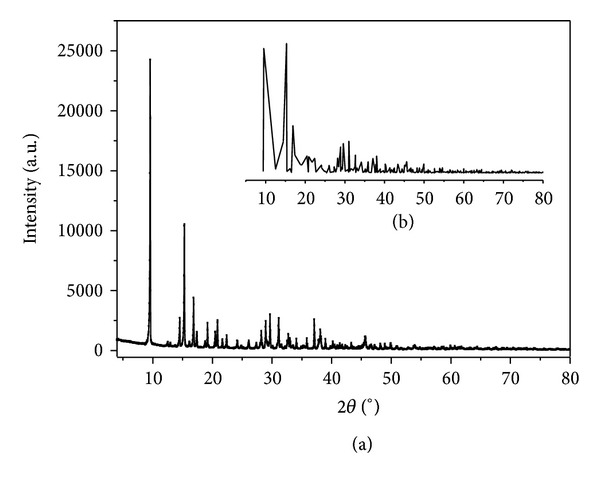
XRD patterns for title complex generated from the experimental data (a) and simulated from the single crystal X-ray data (b).

The X-ray powder diffraction data of [Sb(Hedta)]·2H_2_O was collected in the diffraction angle range of 3°–80°. The XRD pattern of the complex is shown in Figure 4(a). The main diffraction peaks appear at 2*θ* = 9.56°, 15.30°, and 16.85° for the title complex. The index calculation of the XRD data bases on the computer program of least squares method [29] and the calculated results are shown in Table 4. Table 4 shows that the calculated spacing *d*_*hkl*_ is consistent with the experimental ones, and the largest relative deviation between the experimental and calculated spacing *d*_*hkl*_ is less than 0.3%. This indicates that the resultant is a single phase compound [17]. The crystal structure of the complex belongs to an orthorhombic system with the lattice parameters: *a* = 18.479 Å, *b* = 10.957 Å, and *c* = 7.343 Å, respectively. The results of indexes to the XRD data for the complex are consistent with the results of single crystal. The experimental pattern exhibiting some peaks is slightly broadened in comparison with the simulated pattern in Figure 4(b), which may be due to the preferred orientation of the powder samples. The experimental XRD pattern agrees well with the simulated pattern generated on the basis of the single crystal analyses for the title complex.

### 3.3. FT-IR Spectra

**Figure 5:**
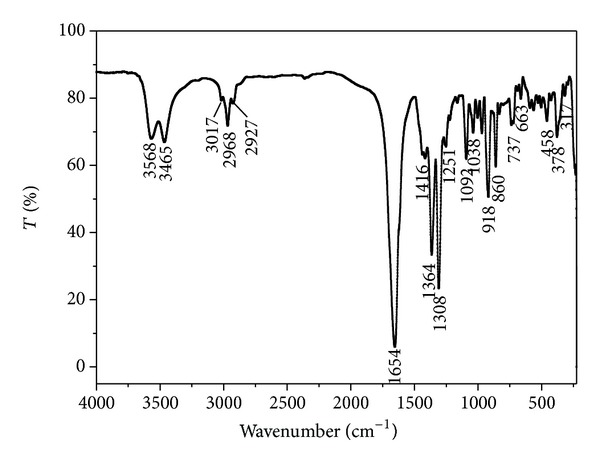
FT-IR spectra of the title complex.

The FT-IR spectra of the title complex are shown in Figure 5. The infrared spectra of the complex show that wide absorption bands in the region 3400–3600 cm^−1^ can be assigned to the O–H stretching vibration of the water molecules. The peaks observed from 3017 to 2927 cm^−1^ are in good agreement with the C–H vibrations. If the band at 1700–1750 cm^−1^ shows the existence of a free carboxyl group, while at about 1650 cm^−1^, suggests that carboxyl groups are coordinated. In which, the *ν*_as_(COO) band of the complex [Sb(Hedta)]·2H_2_O appears at 1654 cm^−1^, both 1364 cm^−1^ and 1308 cm^−1^ are the *ν*_s_(COO) bands The difference value of 290 cm^−1^ and 346 cm^−1^ between the asymmetric and symmetric stretching vibration of the carboxylate group is in line with a monodentate type of coordination [29]. In addition, 1092 cm^−1^ is the specific absorption peak about the C–N vibrations. Compared with the Na_2_H_2_edta ligand, the C–N at 1137 cm^−1^ shifts towards lower frequencies, and it confirms that the nitrogen atoms of the ligand are coordinated to the Sb(III) ion. In the far-infrared region, the absorption peak found in the 378 cm^−1^ region is assigned to the *ν*(Sb–N) vibration and in the 317 cm^−1^ region is assigned to the *ν*(Sb–O) stretching vibration.

### 3.4. Thermal Analysis

**Figure 6:**
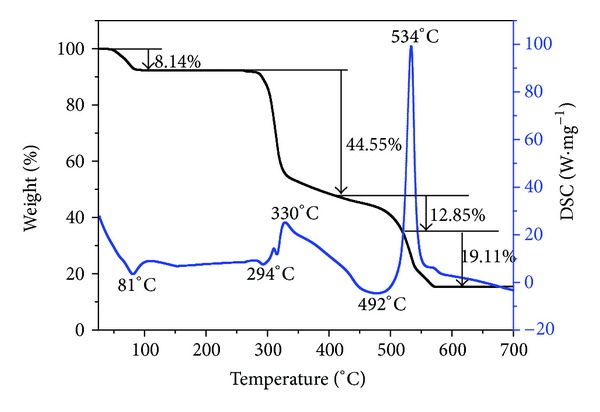
TG-DSC curves of the title complex.

**Scheme 1:**
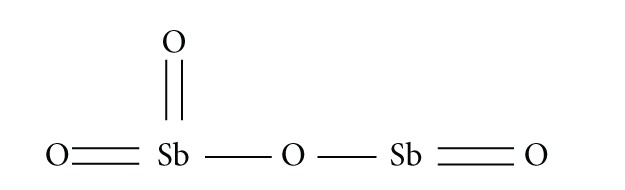
Structure of the Sb_2_O_4_.

The thermal decomposition process of complexes can help us understand the coordination structure of the complexes [30]. The TG-DSC curves of the title complex are given in Figure 6, and the possible pyrolysis reaction and the experimental and calculated percentage mass losses in the thermal decomposition process of the complex are summarized in Table 5. The first mass loss of the complex [Sb(Hedta)]·2H_2_O occurs about 81°C in DSC curve, corresponding to the release of two molecules in crystalline water. This is consistent with the single crystal structure. The experimental percentage mass loss (8.14%) is close to the calculated one (8.06%). Then, a small endothermic peak in DSC curve appears at 294°C. Because there is not any corresponding mass loss of the sample in the TG curve, it can be attributable to structure rearrangement or phase transformation in the solid complex. Thereafter, the exothermic peak at 330°C corresponds to oxidation and decomposition of the ligand, and the experimental mass loss (44.55%) is close to the calculated one (44.57%). This step decomposition product is Sb_2_(CO_3_)_3_. Then, Sb_2_(CO_3_)_3_ is decomposed into Sb_2_O_4_ (Scheme 1), and the experimental and theoretical mass losses are 12.85% and 12.98%, respectively. This is why there is an appreciable endothermic peak at 492°C in DSC curve. However, some of the compounds of antimony may be volatile at high temperature. The final step of the exothermic peak appears at 534°C in DSC curve. The Sb–O single bond is ruptured, and then half of the antimony compound may be oxidized and volatilized. The final residue is antimonous oxide, and the experimental result (15.35%) is in agreement with the result of theoretical calculation (16.30%).

## 4. Conclusion

The complex [Sb(Hedta)]·2H_2_O was synthesized with the reaction of ethylenediaminetetraacetic acid and antimonous oxide as the reactants. The composition and structure of the complex were characterized by EA, singlerystal X-ray diffraction, XRD, FTIR, and TG-DSC. The crystal structure of the complex belongs to orthorhombic system, space group Pna2(1), with cell parameters of *a* = 18.4823(18) Å, *b* = 10.9408(12) Å, *c* = 7.3671(5) Å, *Z* = 4, and *D*_*c*_ = 1.993 g cm^−3^. Sb(III) ion is five-coordinated by two amido N atoms and three carboxyl O atoms from a single Hedta^3−^ ligand, forming a distorted trigonal bipyramid geometry.

## 5. Extra Material

Crystallographic data for the title complex [Sb(Hedta)]·2H_2_O has been deposited with the Cambridge Crystallographic Data Centre. The deposition number is CCDC-953660. The data can be obtained free of charge on application to the Director, CCDC, 12 Union Road, Cambridge, CB2 1EZ, UK, fax: +44 1223 366 033, e-mail: deposit@ccdc.ac.uk, or on the web: www: http://www.ccdc.cam.ac.uk, or from the authors up on request.

## Figures and Tables

**Table 1:** Crystal data and structure refinement for the title complex.

Empirical formula	SbC_10_H_17_N_2_O_10_	*F*(000)	888
Formula weight	447.01	Crystal size	0.46 mm × 0.40 mm × 0.35 mm
Wavelength	0.71073 Å	Theta range for data collection	2.89–25.02°
Temperature	298(2) K	Limiting indices	−21 ≤ *h* ≤ 16, −13 ≤ *k* ≤ 13, −7 ≤ *l* ≤ 8
Crystal system	Orthorhombic	Reflections collected/unique	7102/2529 [*R*(int) = 0.0461]
Space group	Pna2(1)	Completeness to theta = 25.02	99.9%
Unit cell dimensions		Absorption correction	Semiempirical from equivalents
*a*	18.4823(18) Å	Max. and min. transmission	0.5547 and 0.4738
*b*	10.9408(12) Å	Refinement method	Full-matrix least-squares on *F* ^2^
*c*	7.3671(5) Å	Date/restraints/parameters	2529/1/208
*β*	90.00°	Goodness-of-fit on *F* ^2^	1.040
*V*	1489.7(2) Å^3^	Final *R* indices [*I* > 2*σ*(*I*)]	*R*_ 1_ = 0.0332, *wR*_2_ = 0.0811
*Z*	4	*R* indices (all data)	*R*_ 1_ = 0.0359, *wR*_2_ = 0.0832
Calculated density	1.993 g cm^−3^	Absolute structure parameter	−0.27(4)
Absorption coefficient	1.909 mm^−1^	Largest diff. peak and hole	0.786 and −0.805 e Å^−3^

**Table 2:** Selected bond lengths (Å) and angles (°) for the title complex.

Sb(1)–O(1)	2.182(4)	O(7)–C(9)	1.218(7)	N(1)–Sb(1)–N(2)	75.9(2)
Sb(1)–O(5)	2.199(4)	O(8)–C(9)	1.299(7)	C(4)–N(1)–Sb(1)	107.5(3)
Sb(1)–O(3)	2.243(4)	O(1)–Sb(1)–O(5)	142.8(2)	C(6)–N(1)–Sb(1)	108.6(4)
Sb(1)–N(1)	2.336(5)	O(1)–Sb(1)–O(3)	104.36(16)	C(1)–N(1)–Sb(1)	109.7(3)
Sb(1)–N(2)	2.402(5)	O(5)–Sb(1)–O(3)	85.38(15)	C(8)–N(2)–Sb(1)	104.8(3)
O(1)–C(3)	1.307(8)	O(1)–Sb(1)–N(1)	74.09(16)	C(2)–N(2)–Sb(1)	108.5(3)
O(2)–C(3)	1.222(7)	O(5)–Sb(1)–N(1)	75.60(18)	C(10)–N(2)–Sb(1)	114.9(4)
O(3)–C(5)	1.273(7)	O(3)–Sb(1)–N(1)	71.03(17)	C(3)–O(1)–Sb(1)	120.0(4)
O(4)–C(5)	1.229(7)	O(1)–Sb(1)–N(2)	78.88(16)	C(7)–O(5)–Sb(1)	118.9(4)
O(5)–C(7)	1.286(7)	O(5)–Sb(1)–N(2)	73.26(17)	C(5)–O(3)–Sb(1)	120.1(4)
O(6)–C(7)	1.238(7)	O(3)–Sb(1)–N(2)	144.17(16)		

**Table 3:** Hydrogen bond lengths (Å) and bond angles (°) for the title complex.

D–H	*d*(D–H)	*d*(H*⋯*A)	*d*(D*⋯*A)	∠DHA	A symmetry operation
O8–H8	0.820	1.742	2.556	171.90	O9
O9–H9C	0.850	1.854	2.702	175.29	O4 $\left[-x+\frac{1}{2},y+\frac{1}{2},z+\frac{1}{2}\right]$
O9–H9D	0.850	1.935	2.783	175.28	O6 $\left[-x+1,-y+1,z+\frac{1}{2}\right]$
O10–H10E	0.850	2.110	2.950	169.24	O9
O10–H10F	0.850	2.091	2.930	169.18	O2 $\left[-x+\frac{1}{2},y-\frac{1}{2},z+\frac{1}{2}\right]$

**Table 4:** Experimental data and calculated results for powder X-ray diffraction pattern of the complex [Sb(Hedta)]·2H_2_O (orthorhombic system: *a* = 18.479 Å, *b* = 10.957 Å, and *c* = 7.343 Å).

No.	2*θ* (°)	*h*	*k*	*l*	*d*_exp⁡_ (Å)	*d*_cal_ (Å)	*I*/*I*_0_
1	9.56	2	0	0	9.242	9.240	100.0
2	12.52	2	1	0	7.064	7.063	1.5
3	12.96	1	0	1	6.827	6.824	1.2
4	14.51	0	1	1	6.100	6.100	9.5
5	15.30	1	1	1	5.788	5.792	42.6
6	16.17	0	2	0	5.477	5.478	1.6
7	16.85	1	2	0	5.256	5.253	17.2
8	17.41	2	1	1	5.090	5.091	5.2
9	18.11	2	2	0	4.713	4.712	2.1
10	19.19	4	0	0	4.621	4.620	8.5
11	20.48	3	1	1	4.332	4.334	5.5
12	20.85	4	1	0	4.257	4.257	9.7
13	21.69	3	2	0	4.094	4.094	3.0
14	22.40	2	2	1	3.966	3.966	4.5
15	24.15	4	1	1	3.682	3.683	2.7
16	24.86	1	3	0	3.579	3.583	0.9
17	26.10	2	0	2	3.411	3.412	2.8
18	27.37	2	1	2	3.256	3.258	1.8
19	28.21	3	0	2	3.160	3.154	5.9
20	28.95	6	0	0	3.081	3.080	9.4
21	29.69	1	2	2	3.007	3.009	11.8
22	31.12	4	0	2	2.871	2.874	10.6
23	31.61	5	2	1	2.828	2.828	1.3
24	32.73	3	2	2	2.733	2.733	5.0
25	33.00	1	4	0	2.712	2.710	3.6
26	33.54	4	3	1	2.670	2.669	1.1
27	34.10	2	4	0	2.627	2.626	3.4
28	35.24	1	4	1	2.544	2.542	1.1
29	35.84	3	4	0	2.503	2.503	3.5
30	37.07	1	0	3	2.423	2.426	10.2
31	37.80	7	2	0	2.378	2.378	3.0
32	38.11	2	0	3	2.360	2.366	6.7
33	38.95	2	1	3	2.311	2.313	3.4
34	39.81	4	3	2	2.262	2.259	0.8
35	40.18	4	4	1	2.242	2.244	2.5
36	40.50	1	2	3	2.225	2.219	1.2
37	40.95	0	4	2	2.202	2.196	0.9
38	41.39	2	2	3	2.178	2.172	1.7
39	41.77	4	0	3	2.161	2.163	1.2
40	42.28	7	0	2	2.137	2.143	1.1
41	43.31	1	5	1	2.087	2.086	1.9
42	45.19	4	2	3	2.006	2.012	1.3
43	45.69	2	3	3	1.984	1.986	4.1
44	46.62	8	3	0	1.947	1.952	1.3
45	47.18	3	3	3	1.926	1.931	1.2
46	48.17	5	4	2	1.888	1.888	1.8
47	48.93	4	3	3	1.860	1.861	1.8
48	49.89	0	4	3	1.826	1.825	1.9
49	50.90	2	4	3	1.792	1.791	1.0
50	53.92	4	4	3	1.698	1.697	1.3
51	58.45	3	6	2	1.578	1.580	0.8
52	58.77	0	7	0	1.569	1.565	0.8
53	59.94	2	7	0	1.542	1.543	1.2
54	60.66	0	4	4	1.525	1.525	1.1
55	61.79	5	3	4	1.500	1.499	0.9
56	64.46	2	1	5	1.442	1.438	0.9
57	67.57	2	5	4	1.388	1.391	0.9

**Table 5:** Thermal decomposition data of the title complex.

Reaction	DSC (°C)	Mass loss (%)	
		*W*_exp⁡_	*W*_theor_
[Sb(Hedta)]·2H_2_O			
↓ −2H_2_O	81 (endo.)	8.14	8.06
[Sb(Hedta)]			
↓ structural rearrangement	294 (endo.)		
[Sb(Hedta)]			
↓ −C_8.5_H_13_N_2_O_3.5_	330 (exo.)	44.55	44.57
1/2Sb_2_(CO_3_)_3_			
↓ −C_3_O_5_	492 (exo.)	12.85	12.98
1/2Sb_2_O_4_			
↓ −Sb_0.5_O_1.25_	534 (endo.)	19.11	18.09
1/4Sb_2_O_3_		15.35^a^	16.30^b^

^a^The experimental percentage mass of the residue in the sample; ^b^the calculated percentage mass of the residue in the sample.
